# Photodynamic therapy with talaporfin sodium for endoscopically unresectable gastric cancer using a novel simultaneous light‐emitting method

**DOI:** 10.1002/deo2.334

**Published:** 2024-01-23

**Authors:** Tomoyuki Hayashi, Kotaro Hayashi, Takeshi Terashima, Masaki Nishitani, Noriaki Orita, Masaki Miyazawa, Akihiro Seki, Hidetoshi Nakagawa, Kouki Nio, Noriho Iida, Shinya Yamada, Hajime Takatori, Tetsuro Shimakami, Taro Yamashita

**Affiliations:** ^1^ Department of Gastroenterology Kanazawa University Hospital Ishikawa Japan

**Keywords:** endoscopic submucosal dissection, gastric cancer, photodynamic therapy, simultaneous light‐emitting method, talaporfin sodium

## Abstract

We describe a case of gastric cancer treated by photodynamic therapy (PDT) with talaporfin sodium using a novel simultaneous light‐emitting method. An 82‐year‐old man was diagnosed with gastric cancer near the cardia with suspected deep submucosal invasion. Surgical resection was deemed high‐risk owing to an underlying pulmonary disease. After ruling out endoscopic procedures due to intense fibrosis resulting from the scarring, PDT with talaporfin sodium was chosen. PDT was successfully conducted using an endoscope with simultaneous light emission. The patient experienced a complete response to the treatment and showed no signs of recurrence during follow‐up. This case highlights the potential of PDT with talaporfin sodium as a viable alternative for challenging cases, particularly in patients unsuitable for surgery and endoscopic resection. Furthermore, the novel simultaneous light‐emitting method may improve the efficiency of the procedure. This case demonstrates the potential of PDT in gastric cancer treatment, especially for high‐risk patients.

## INTRODUCTION

Photodynamic therapy (PDT) involves the selective elimination of cancer tissues through light‐triggered chemical reactions. A second‐generation photosensitizer, talaporfin sodium, has shown promise owing to its ability to shorten the duration of hospitalization and light avoidance. This approach is especially beneficial for recurrent or inoperable lesions. However, PDT for gastric cancer is currently less accepted. We report a case of gastric cancer treated by PDT with talaporfin sodium using a novel simultaneous light‐emitting method. A combination of talaporfin sodium and the simultaneous light‐emitting endoscope showed significant potential for improved treatment outcomes of PDT.

### Case report

**FIGURE 1:**
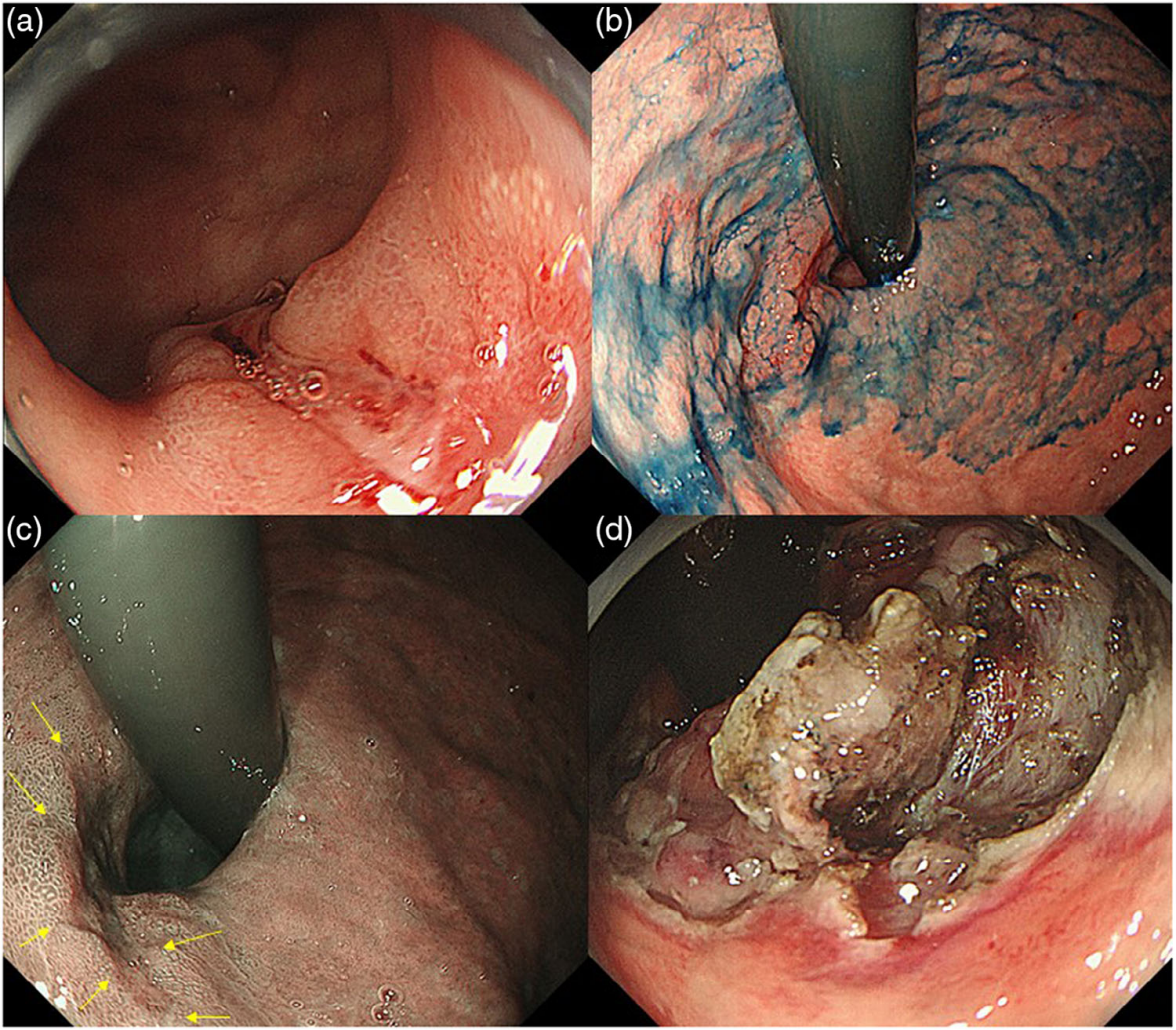
(a, b) Esophagoduodenogastroscopy revealed an early gastric cancer with suspected deep submucosal invasion and scarring observed just below the gastric cardia. (c) A demarcation line was clearly visible in narrow‐band imaging endoscopy. (d) During the course of the endoscopic submucosal dissection, the resection was discontinued due to intense fibrosis resulting from the scarring.

An 82‐year‐old man previously underwent endoscopic submucosal dissection (ESD) for early gastric cancer on the posterior wall of the upper gastric body at another hospital 2 years ago. During postoperative follow‐up esophagoduodenogastroscopy, early gastric cancer with suspected deep submucosal invasion and scarring was observed just below the gastric cardia (Figure 1).

Although surgical resection is the first treatment of choice when lymph node metastasis cannot be ruled out, the patient, on evaluation by an anesthesiologist was found to have chronic obstructive pulmonary disease and post‐radiotherapy status for lung cancer, with progressive deterioration in lung function, making general anesthesia exceedingly risky. Therefore, despite the potential risk of lymph node metastasis, ESD was opted for. Difficulties were encountered during the procedure due to intense fibrosis resulting from the scarring, and the resection was discontinued. Subsequently, the patient was referred to our hospital to seek another treatment.

**FIGURE 2:**
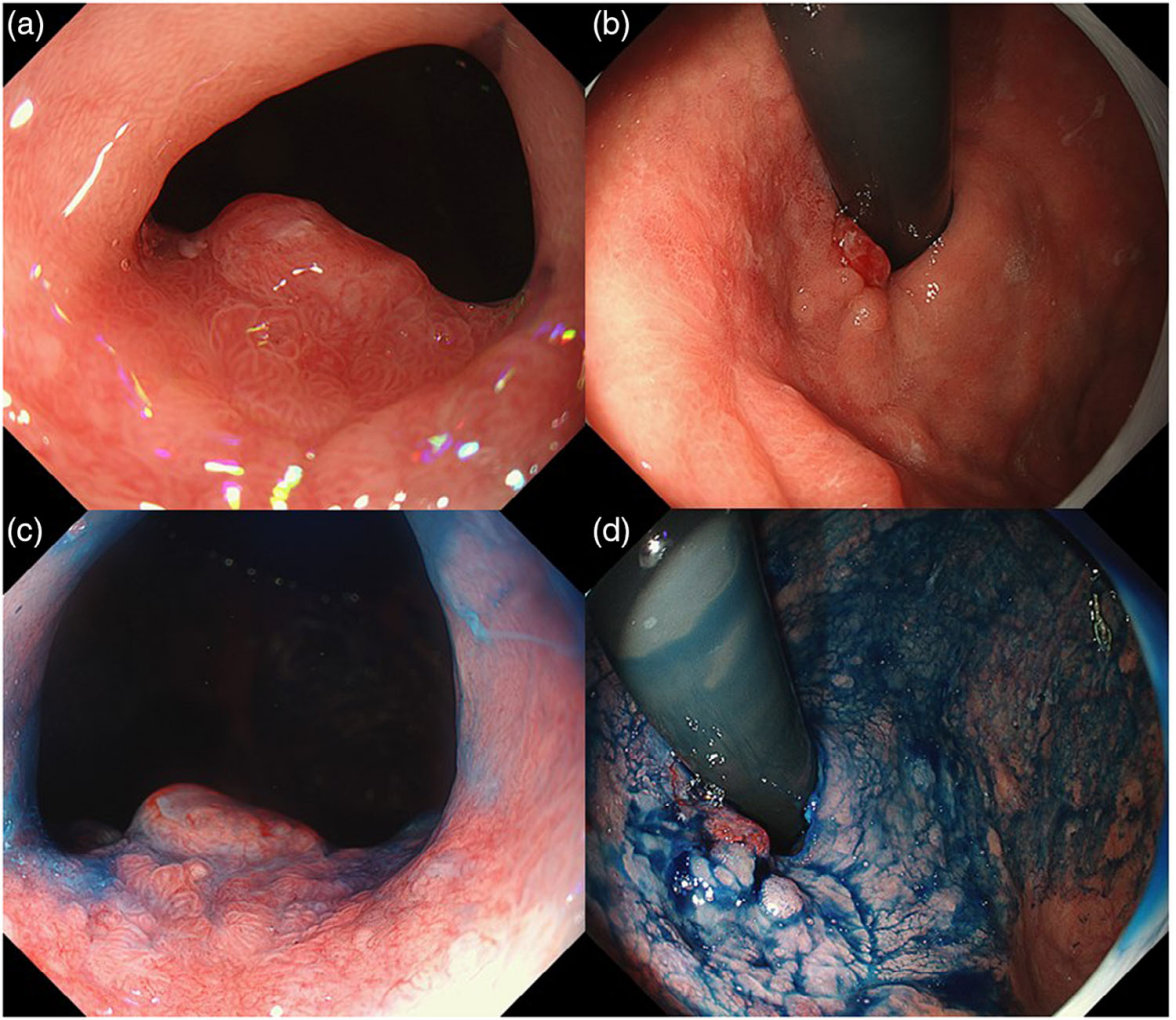
Esophagoduodenogastroscopy after endoscopic submucosal dissection failure revealed a reddish flat and elevated lesion with a surrounding mucosal contraction located just below the greater curvature of the gastric cardia.

On evaluation after ESD failure, the lesion appeared reddish, flat, and elevated with a surrounding mucosal contraction located just below the greater curvature of the gastric cardia (Figure 2). Histologically, a regional increase in glandular density, cells with an increased nucleus/cytoplasm ratio, and multi‐layered mitotic figures were detected, leading to a diagnosis of well‐differentiated adenocarcinoma. As over a month had elapsed since the previous ESD attempt at the other hospital, ESD was anticipated to be challenging. Our anesthesiologist also assessed that general anesthesia would pose an elevated risk owing to the patient's compromised respiratory function. Given our experience with salvaging PDT with talaporfin sodium (Laserphyrin; Meiji Seika Pharma) for esophageal cancer, we opted to proceed with PDT with talaporfin sodium.

As PDT employing talaporfin sodium for gastric cancer is not currently approved under Japanese medical insurance, we applied for and subsequently received approval for this treatment from the Department of New Medical Technology Management at Kanazawa University Hospital.

**FIGURE 3:**
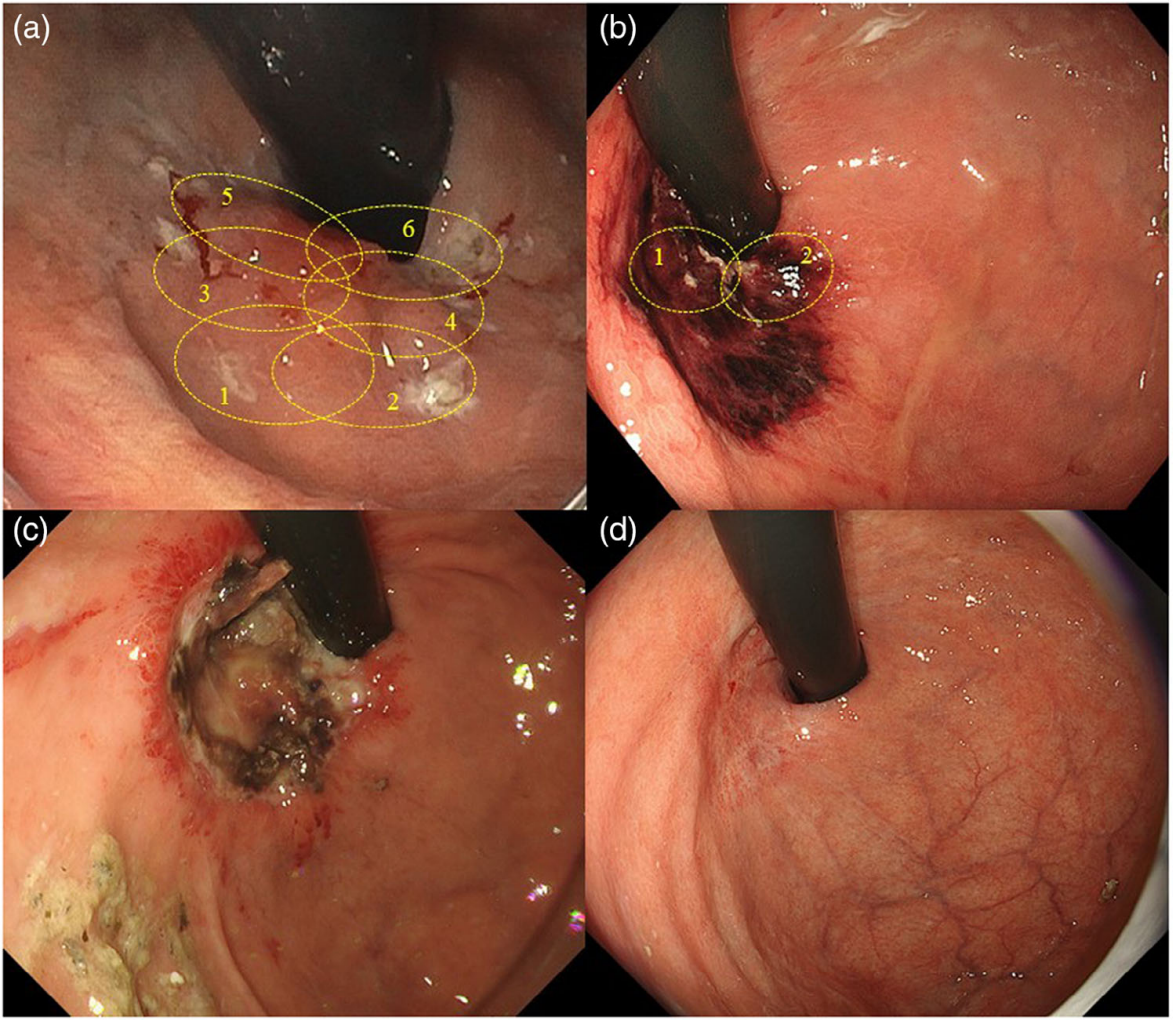
(a) The area was exposed to an overall laser dose of 600 J. (b) On the following day, we observed ischemic change almost throughout the entire lesion. The area where ischemic change was thought to be relatively scant was subjected to an additional 200 J irradiation (total 800 J). (c) After 7 days of treatment, the irradiated area was ulcerated. (d) Esophagoduodenogastroscopy 3 months after treatment revealed that the lesion had completely transformed into scar tissue, and biopsy results showed the absence of cancer cells, which indicated a local complete response.

After obtaining consent from the patient, PDT was performed with an endoscope using a simultaneous light‐emitting method (GIF‐H190; Olympus Medical Systems). The video endoscopy system comprised a video processor (EVIS EXERAIII CV‐190PLUS; Olympus Medical Systems) and a light source (EVIS EXERAIII CLV‐190; Olympus Medical Systems). A disposable hood (Elastic Touch; Top Corporation) was attached to the distal tip of the endoscope to ensure an unobstructed field of view. Five hours following the intravenous administration of 40 mg/m^2^ talaporfin sodium, a diode laser (PD laser; Meiji Seika Pharma) was used to irradiate the lesion at 664 nm. The patient underwent four sessions of 100 J each to the anal side of the lesion using an inverted endoscope procedure, and the remaining area on the oral side was subsequently irradiated in two sessions in a look‐down procedure from the esophageal side. During the inverted endoscope procedure, the probe was passed through the forceps hole of the endoscope without resistance, the probe was not damaged, and the laser dose was not reduced. Consequently, the lesion was exposed to an overall laser dose of 600 J (Figure 3a). On the following day, we observed ischemic change throughout almost the entire lesion. The area where the ischemic change appeared relatively scant was subjected to an additional 200 J irradiation to achieve a total dose of 800 J (Figure 3b). The endoscope insertion time on the treatment and following days was 95 and 35 min, respectively. After 7 days of treatment, the irradiated area was ulcerated (Figure 3c). The patient was discharged 8 days after treatment because no skin symptoms were observed after a 5‐min light‐exposure test and no adverse effects such as aspiration pneumonia developed. At 1 month after treatment, scarring was in progress. Esophagoduodenogastroscopy 3 months after treatment showed that the lesion had completely transformed into scar tissue, and biopsy results showed an absence of cancer cells, suggesting a local complete response (Figure 3d). In over a year following treatment, there has been no recurrence.

## DISCUSSION

PDT has been established as a secure approach for treating conditions characterized by abnormal tissue growth, with a prominent focus on its application in cancer therapy. The antitumor effects are influenced by factors such as vascular shutdown, inflammation, and antitumor immunity.^1^ PDT involves the use of photosensitizers such as porfimer sodium, which possesses a propensity for accumulating within tumor tissues and sites of neovascularity. This process is then activated by a low‐power red light.^2^ Clinical trials of PDT for early gastric cancer showed excellent curative effects. In studies on PDT for gastric cancer, 77.3%–88% of patients achieved a complete response.^3^, ^4^, ^5^ PDT for early gastric cancer was incorporated into the Japanese Universal Health Insurance Coverage System in 1996. However, a more curative resection technique known as ESD emerged and gained widespread adoption. Owing to its advantage of allowing a comprehensive pathological examination, the Japanese Gastric Cancer Association's guidelines designated endoscopic resection as the recommended standard treatment for cancers without lymph node metastasis. Consequently, PDT was omitted from the guidelines and has not yet gained widespread acceptance.^6^

PDT can be challenging for patients owing to the need for an extended period of light avoidance, which can result in significant inconvenience and a decline in cognitive function. A second‐generation photosensitizer, talaporfin sodium is rapidly cleared from the skin and requires a shorter hospitalization and light avoidance period (≤2 weeks).^7^ Its effect is expected to reach a deeper layer in the muscularis propria because the diode laser's excitation wavelength is longer than that of an excimer dye laser with porfimer sodium. Additionally, the diode laser is smaller and less expensive than the excimer dye laser.^8^ PDT with talaporfin sodium is an excellent option for recurrent or residual lesions following endoscopic therapy and in cases of inoperability owing to the patient's age nutritional status or other considerations.^9^ For local failure after radiation therapy for esophageal cancer, a multi‐institutional phase II study revealed that salvage PDT with talaporfin sodium achieved an 88.5% complete response rate.^10^ For gastric cancer, an investigator‐initiated clinical trial of PDT with talaporfin sodium was planned; however, no results were reported as of November 2023. Few English case reports discuss PDT for gastric cancer with talaporfin sodium. This case report is clinically significant, highlighting reduced hospitalization and light avoidance periods with talaporfin sodium compared to porfimer sodium.

**FIGURE 4:**
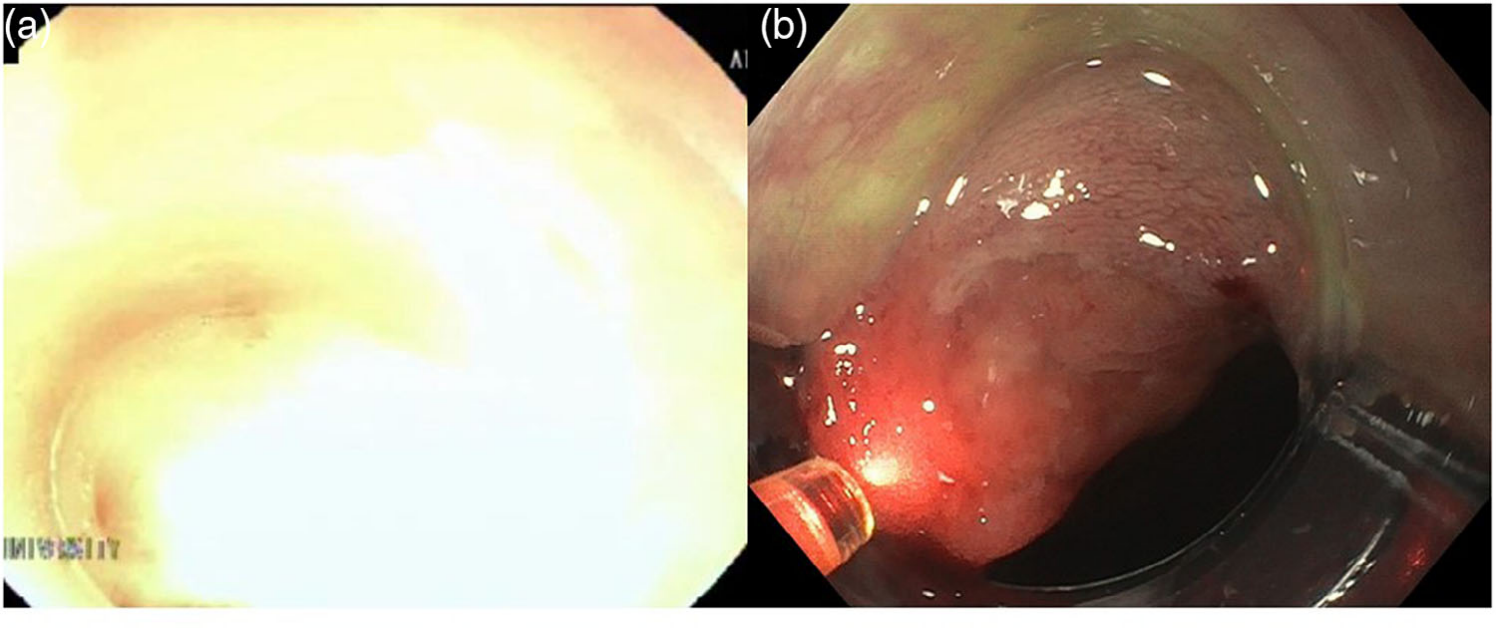
(a) In previous endoscope systems (EG‐600WR), the light intensity of the laser on the screen was too strong. (b) Because the light intensity of the laser on the screen of a novel endoscope (GIF‐H190) with simultaneous light emission (EVIS EXERAIII CLV‐190) is not too bright, it is possible to easily detect the lesion.

When a monochromatic laser is used with an endoscope employing a frame sequential method, the laser is perceived as a strong white light, consequently losing color information in the endoscopic image, rendering it unsuitable for PDT. Therefore, PDT requires an endoscope using a simultaneous light‐emitting method. Previously, the light intensity of the laser on the screen was so strong (Figure 4a) that discerning whether the irradiated point had shifted due to body movement, coughing, or other factors was often impossible. Such a shift required interruption of the treatment, and readjustment of the endoscope to the proper position, significantly lengthening treatment time. Because the laser's light intensity when using a simultaneous light‐emitting method is not too bright, lesions are easier to detect (Figure 4b) even if the field of view has shifted, thereby drastically shortening treatment time and reducing irradiation of non‐lesion areas.

Although PDT for gastric cancer is not yet widely accepted, PDT employing talaporfin sodium has mitigated drawbacks such as the need for prolonged light shielding, and is emerging as a favorable treatment alternative, especially for elderly patients facing the challenges of endoscopic or surgical resection. The simultaneous light‐emitting method has alleviated the complexities of PDT, and this approach has the potential to become a viable treatment for gastric cancer.

## CONFLICT OF INTEREST STATEMENT

None.
